# Evaluating the presence of *Mycoplasma hyorhinis*, *Fusobacterium nucleatum,* and *Helicobacter pylori* in biopsies of patients with gastric cancer

**DOI:** 10.1186/s13027-021-00410-2

**Published:** 2021-12-23

**Authors:** Camila do Nascimento Araujo, Aline Teixeira Amorim, Maysa Santos Barbosa, Julieta Canjimba Porto Lucas Alexandre, Guilherme Barreto Campos, Cláudia Leal Macedo, Lucas Miranda Marques, Jorge Timenetsky

**Affiliations:** 1 Department of Microbiology, Institute of Biomedical Sciences, ICB/USP, University of São Paulo, São Paulo, Brazil; 2 Department of Animal Health, Faculty of Veterinary Medicine, José Eduardo dos Santos University, Huambo, Angola; 3 Multidisciplinary Health Institute /Campus Anísio Teixeira, IMS/CAT – UFBA, Federal University of Bahia, Vitória da Conquista, Brazil; 4 Micro – Pathological Anatomy and Cytopathology Service, Vitória da Conquista, Brazil

**Keywords:** Mollicutes, *Mycoplasma hyorhinis*, *Fusobacterium nucleatum*, *Helicobacter pylori*, Gastric cancer

## Abstract

**Background:**

Gastric cancer is the third leading cause of cancer-related deaths worldwide and has been associated with infections that may promote tumour progression. Accordingly, we analysed the presence of Mollicutes, *Mycoplasma hyorhinis*, *Fusobacterium nucleatum* and *Helicobacter pylori* in gastric cancer tissues and evaluated their correlation with clinicopathological factors.

**Methods:**

Using a commercial kit, DNA were extracted from 120 gastric samples embedded in paraffin: 80 from patients with gastric cancer and 40 from cancer free patients, dating from 2006 to 2016. *Mollicutes* and *H. pylori* were detected by PCR; *F. nucleatum* and *M. hyorhinis* were detected by qPCR, together with immunohistochemistry for the latter bacteria.

**Results:**

Mollicutes were detected in the case and control groups (12% and 2.5%) and correlated with the papillary histologic pattern (*P* = 0.003), likely due to cell transformation promoted by Mollicutes. *M. hyorhinis* was detected in the case and control group but was not considered a cancer risk factor. *H. pylori* was detected at higher loads in the case compared to the control group (8% and 22%, *P* = 0.008) and correlated with metastasis (*P* = 0.024), lymphatic invasion (*P* = 0.033), tumour of diffused type (*P* = 0.028), and histopathological grading G1/G2 (*P* = 0.008). *F. nucleatum* was the most abundant bacteria in the case group, but was also detected in the control group (26% and 2.5%). It increased the cancer risk factor (*P* = 0.045, OR = 10.562, CI95% = 1.057–105.521), and correlated with old age (*P* = 0.030) and tumour size (*P* = 0.053). Bacterial abundance was significantly different between groups (*P* = 0.001).

**Conclusion:**

Our findings could improve the control and promote our understanding of opportunistic bacteria and their relevance to malignant phenotypes.

## Background

The mortality rates due to neoplasia exceed those caused by coronary heart disease or stroke, and the highest rates are reported in low- to middle-income households [1]. Globally, gastric cancer is the fifth most diagnosed and the third leading cause of cancer-related deaths that mainly affects men over 50 years of age. Its prevalence and prognosis vary considerably between racial and socioeconomic groups. In Brazil, mortality rates due to gastric cancer rank fourth among men and sixth among women [1, 2]. Gastric cancer affects different sites in the stomach with macroscopic variations and distinct histological patterns. According to Lauren's classification, stomach cancer can be divided into intestinal type and diffuse type [3]. The development of stomach tumours has primarily been associated with an individual's genetics, environmental factors, and infection with pathogens that induce persistent inflammation. For example, *Helicobacter pylori* is a pathogen that induces peptic ulcers, neutralises gastric acid, and, with their *cag* pathogenicity island, maintains a tumour-permissive microenvironment in the gastric mucosa [4].

Opportunistic microorganisms, such as Mollicutes and *Fusobacterium nucleatum*, are not considered to be type I carcinogens. However, given their prevalence in tumour tissues, their potential roles as oncobacteria deserve investigation. *F. nucleatum*, a Gram-negative, anaerobic, biofilm-forming, non-spore-forming and non-mobile bacillus, occurs at equilibrium in the oral-gastrointestinal tracts and participates in the health and homeostasis of the oral site [5]. *F. nucleatum* has also been associated with extraoral diseases such as appendicitis and intestinal inflammation [5]; has been reported in oral, head and neck, cervical and gastric carcinoma tissues; and is commonly associated with colorectal cancer, where it induces a worse prognosis by promoting resistance to chemotherapy [6, 7]. The association of *F. nucleatum* with malignant lesions can be explained by microsatellite instability, alteration of the Cpg island methylator phenotype (CIMP), and mutations in the BRAF, KRAS, and TP53 genes [8]. *F. nucleatum* can migrate out of the oral site via an oral-gastrointestinal route or haematogenously in a Fap2 adhesin-dependent manner. Fap2 is an adhesin unique to oral *Fusobacteria* that binds to GalGalNac (a sugar residue overexpressed in tumour tissue) or to the T cell immunoglobulin and ITIM domain (TIGIT) (a receptor of natural killer cells and lymphocytes), inhibiting the cytotoxic function of these cells and promoting a conducive environment for bacteria and tumour cells [9].

The human microbiome harbours not only *F. nucleatum*, but also bacteria of the Mollicutes class that may cause or be associated with host immunosuppression [5, 10], and have been detected in tissues of patients with chronic gastritis, local inflammation, and neutrophil accumulation [11]. *Mollicutes* belong to a heterogeneous group of microorganisms that are present in humans, animals, insects, and plants [10]. Most pathogenic species in humans and animals belong to the order *Mycoplasmatales*, with 160 *Mycoplasma* species and 8 *Ureaplasma* species [12]. *Mycoplasma* species, like the porcine *M. hyorhinis,* are common cell culture contaminants. *Mycoplasma hyorhinis* can, reportedly, invade mammalian immune cells and has been detected in gastric carcinoma tissues [13, 14].

*Mycoplasma hyorhinis* has been implicated in several swine diseases [15], with severe pathogenicity, mortality rates, and subsequent economic losses. Attempts to minimise these impacts with antibiotics promote the development of drug resistant *M. hyorhinis* strains, which infect the human stomach via the consumption of porcine products [11, 15]. A previous report identified *M. hyorhinis* in 56% of a gastric cancer [16]. Its association with gastric cancer is more common than with other gastric diseases [17] where it is able to induce different profiles that cause chronic superficial gastritis, gastric ulcers, and intestinal metaplasia [16]. Lipoprotein p37, a component of *M. hyorhinis* that possesses a transport system similar to the periplasmic transport systems of Gram-negative bacteria, is believed to promote cell motility and invasion, consequently exacerbating carcinogenesis and metastasis [18, 19]. These features have been identified both in vitro and in vivo and were associated with the phosphorylation of epidermal growth factor receptor (EGFR) and extracellular signal-regulated kinase 1/2 (ERK1/2) [13]. The EGF binding/receptor system appears to be involved in regulating gastric mucosal proliferation and the progression of gastric carcinomas [20]. *M. hyorhinis* infection, or the presence of p37 alone, might induce the phosphorylation of PI3k and AKT, which are EGFR-dependent. Activation of the EGFR-PIK3-AKT pathway is related to the regulation of metabolism, growth, survival, and cell motility; the dysregulation of this pathway has been linked to tumorigenesis and angiogenesis [21].

Infection with *M. hyorhinis* and the p37 protein can potentially promote gastric cancer development, though the underlying mechanism remains unclear and needs further elucidation [13, 21]. Based on the strong association between gastric cancer and infectious agents, we evaluated the relationship between specific clinical variables and infection with Mollicutes as well as *F. nucleatum* and *H. pylori*. Our study group consisted of individuals with or without gastric cancer from Vitória da Conquista, Bahia (BA), Brazil. Not all samples positive for Mollicutes were positive for *M. hyorhinis*, indicating that other mycoplasma species may be present in the gastric tissue.

## Methods

### Study population

We performed a retrospective study assessing formalin-fixed paraffin-embedded (FFPE) gastric fragment samples collected from 2006 to 2016. These samples were obtained from a laboratory of pathological anatomy and cytopathology service in Vitória da Conquista, BA, Brazil. In total, 120 samples were examined: 80 from patients with gastric cancer (case group) and 40 from patients undergoing stomach reduction surgery, with intact tissue confirmed by histopathology (control group).

### Sample collection

Gastric fragments of specific tumour areas were aseptically obtained from paraffin blocks; the first section from the paraffin block was consistently discarded [22]. Lesions were evaluated according to the surgical classification of the stomach: (a) cardia, (b) body and fundus, and (c) antrum. The molecular diagnosis of FFPE blocks was performed using five 10-μm-thick slices stored in 1.5 mL microtubes. Immunohistochemical (IHC) and histology (haematoxylin–eosin [H&E] staining) analyses were performed using 4-μm-thick sections [23].

### DNA extraction from formalin-fixed paraffin-embedded (FFPE) gastric fragment samples

Due to the difficulties in recovering nucleic acids from FFPE samples and the diversity of protocols available in the literature, we selected an optimal method based on assay duration and the viability of extracted DNA. Four methods were in-house and were assessed with and without prior xylol dewaxing, and two other methods were performed using commercial kits and were tested with and without prior xylol and mineral oil dewaxing. After dewaxing the samples, the extraction methods were tested with different digestion buffers, as described:

- *Dewaxing with Xylol* [24]: This was performed for in-house methods before DNA extraction. In brief, DNA samples were immersed in xylol (pH 6–7.6) at 65 °C for 10 min and then bathed in 99°, 96°, and 70° Gay-Lussac (GL) and volatilised for 30 min.
- *Dewaxing with Mineral Oil* [25]: This was performed before DNA extraction using commercial kits. Briefly, DNA samples were immersed in mineral oil, incubated for 1 min at 80 °C, and vortexed.
- *Extraction Method 1 (EM1)*: digestion buffer (pH 7.5), with 1 M Tris–HCl, 1% SDS (sodium dodecyl sulphate), and PK (proteinase K) (10 mg/mL) [24].
- *Extraction Method 2 (EM2)*: digestion buffer (pH 8.0), with 1 M NaCl, 1 M Tris–HCL, 0.5 M EDTA, 10% SDS, and PK (0.5 mg/mL) [26].
- *Extraction Method 3 (EM3)*: digestion buffer (pH 8.5), with 50 mM Tris–HCL, 1 mM EDTA, and 0.5% Tween 20 and PK (200 μg/mL) [16].
- *Extraction Method 4 (EM4)*: digestion buffer (pH 9.0), with 50 mM Tris–HCl and PK (20 mg/mL) [27].

Subsequently, samples were incubated at 95 °C for 10 min, purified using a solution of phenol/chloroform/isoamyl alcohol (25:24:1; pH 8.0), treated with ammonium acetate (7.5 M), precipitated with ice-cold ethanol followed by alcohol baths, and volatilised for 30 min [28]. Samples were homogenised with 40 μL of Tris–EDTA buffer (10 mM Tris–HCl, 1 mM EDTA, pH 8.0) and stored at − 20 °C.

- Extraction Method 5 (EM5): after dewaxing, DNA extraction was performed using a ReliaPrep™ FFPE gDNA Miniprep System (Promega®, Madison, WI, USA).
- Extraction Method 6 (EM6): after dewaxing, DNA extraction was performed using a Macherey–Nagel® Tissue Kit (Fisher Scientific®, Fair Lawn, NJ, USA).

For each extraction method, the amount and purity of DNA were assessed spectrophotometrically (Nanospectrometer-DS-11, DeNovix®, Wilmington, DE, USA).

### Molecular detection

#### β-globin PCR

The primers PC03/PC04 for the housekeeping gene (*β-globin*) [29] were standardised to verify the quality of the extracted genetic material. Primer sequences and the expected product size are listed in Table 1. Genomic DNA samples were amplified by PCR, and the product size was verified by performing agarose gel (2%) electrophoresis. DNA derived from frozen tissue and Si-Ha cell culture were used as positive controls, with ultrapure water employed as a negative control. All PCR reactions on FFPE extracted material were performed in duplicate.

**Table 1:** Primers used in the PCR assay for detecting *β-globin*, Mollicutes, *Helicobacter pylori*, *Fusobacterium nucleatum*, and *Mycoplasma hyorhinis*

Primer	Sequence (5′–3′)	Location	Amplicons	Ref
PC03	ACACAACTGTGTTCACTAGC	nt 1575-1594	110 bp	[29]
PC04	CAACTTCATCCACGTTTCAC	nt 1684-1669		
GPO3	GGGAGCAAACACGATAGATACCCT	nt 221-245	270 bp	[32]
MGSO	TGCACCATCTGTCACTCTGTTAACCTC	nt 491-465		
JW21	GCGACCTGCTGGAACATTAC	nt 691-710	138 bp	[33]
JW22	CGTTAGCTGCATTACTGGAGA	nt 829-809		
FNF	CAACACCTAGTAATCATC	nt 2.443.126-2.443.126	653 bp	
FNR	CGAATGCTAATACCTATA	nt 2.443.761-2.443.778		[35]
FN-Probe	Cy5-GGCTTCCCCATCGGCATTCC-BHQ	nt 2.443.229-2.443.248	604 bp	[34]
MHRHF	GAACGGGA TGTAGCAATACATTC	nt 18520-18542		
MHRHR	AGCGGACTGAAGTTGAGCTTCAG	nt 19124-19102		
Mhr-p37-RT-F	TATCTCATTGACCTTGACTAAC	nt 768.070-768.092	89 bp	[15]
Mhr-p37-RT-R	ATTTTCGCCAATAGCATTTG	nt 816.070-835.070		
Mhr-p37-RT-Probe	6FAM-CATCCTCTTGCTTGACTACTCCTG-MGBNFQ	nt 774.070-796.070		

Target sequences for *β-globin* amplification – PC03/PC04 (110 bp). Target sequences for 16S rRNA of Mollicutes—GPO3/MGSO (270 bp), *H. pylori*—JW21/JW22 (138 bp), *F. nucleatum*—FNF/FNR (653 bp), *M. hyorhinis*—MHRHF/MHRHR (604 bp), and for p37 gene rRNA of *M. hyorhinis*—Mhr-p37-RT-F/ Mhr-p37-RT-R / Mhr-p37-RT-Probe (89 bp)

To prepare PCR reactions, we used 50 ng/µL of purified DNA from each sample, 1 × buffer (Buffer—10 × Tris–HCl [pH 8.4], 500 mM KCl), 1.6 mM of deoxyribonucleotide triphosphate (dNTP), 1.5 mM of MgCl_2_, 20,000 nM of each primer (Table 1), 0.5 U of Taq DNA polymerase (Invitrogen®, São Paulo, SP, Brazil), to achieve a final volume of 25 µL. The assays were performed in a Veriti Thermal Cycler (Applied Biosystems, São Paulo, SP, Brazil). The reaction parameters were as follows: denaturation of 95 °C for 5 min, 40 cycles at 95 °C for 1 min, with different annealing temperatures tested (54–56 °C) for 1 min, 72 °C for 1 min, a final cycle of 72 °C for 10 min, and a *hold step* at 4 °C.

#### Mollicutes, *M. hyorhinis*, *F. nucleatum*, and *H. pylori* detection

Different strains of mycoplasma were cultured, and their DNA was extracted using a PureLink™ Genomic DNA Mini Kit (Thermo Fisher Scientific, Waltham, MA, USA)*. M. hyorhinis* (ATCC 17981) was cultured in FRISS medium (pH 7.2) [30]; *M. bovis* (ATCC 19210 – AN5) and *M. hominis* (ATCC 23114-PG-21) were cultured in SP4 medium (pH 7.4), supplemented with glucose or arginine [31]. The strains were obtained from the *Mycoplasma* Laboratory collection at the Institute of Biomedical Sciences, University of São Paulo, SP, Brazil.

Conventional PCRs were performed to detect 16S rRNA of Mollicutes [32], *H. pylori* [33], and *M. hyorhinis* [34]*.* Accordingly, 50 ng/µL of purified DNA was used for each sample, 1 × buffer (Buffer—10 × Tris–HCl [ pH 8.4], 500 mM KCl), 0.2 mM of deoxyribonucleotide triphosphate (dNTP), 1.5 mM of MgCl_2_, 50,000 nM of each primer (Table 1), and 1.0 U of Taq DNA polymerase (Invitrogen®), to achieve a final volume of 25 µL. For Mollicutes, genus-specific universal primers were used instead. The assays were performed using a Veriti® Thermal Cycler (Applied Biosystems, São Paulo, SP, Brazil). The parameters for each reaction have been previously described [32–34].

Quantitative PCR was used to detect 16 s rRNA of *F. nucleatum* [35] and p37 of *M. hyorhinis* [15]. First, mycoplasmas were cultured, and the DNA was extracted according to the manufacturer’s instructions. The genomic DNA copy number was then calculated by spectrophotometry. Ten-fold serial dilutions (10^7^–10 copies/μL) of the mycoplasma DNA standard were prepared and analysed. A standard curve was prepared for each reaction. Samples were analysed in triplicate using a 7300 Real-Time PCR System (Applied Biosystems). The reagents used were as follows: 50 ng/µL of DNA extracted by EM6, 12.5 µL of Master Mix (Applied Biosystems), 100 µM of each primer (Table 1), and 0.3 µM Taqman probe (Table 1); the final volume was 25 µL. The samples were amplified using StepOne™ software (Applied Biosystems) with the following cycles: 50 °C for 2 min, 95 °C for 10 min, 40 cycles of denaturation at 95 °C for 15 s, and annealing at 55 °C for 30 s (to detect *F. nucleatum* DNA) or 57 °C for 15 s (to detect *M. hyorhinis* DNA) and extension at 60 °C for 1 min.

### Immunohistochemistry detection

To confirm whether there was a higher charge of *M. hyorhinis* that was not found by molecular methods, 120 4-μm-thick sample sections were assessed for *M. hyorhinis* occurrence using IHC. Si-Ha cells with or without *M. hyorhinis* coinfection were used as positive and negative controls, respectively [36]. Unstained sections of each sample were dewaxed by twice immersing them in xylol baths for 10 min, followed by rehydration in consecutive graduated ethanol baths (99°, 96°, and 70° GL) and distilled water. Peroxidase was blocked with H_2_O_2_ (10 vol.) for 10 min, twice, and the samples were then washed with distilled water. Antigenic recovery was carried out in an electric pressure cooker for 15 min using Trilogy™ Cell Marque solution, followed by washing with distilled water and Tris Buffered Saline (TBS; pH 7.4). The slides were incubated overnight with primary horse polyclonal antibodies against *M. hyorhinis*-7 (NIAID, Bethesda, MD; 1:100) in a darkroom at 4 °C. Next, slides were washed three times using TBS for 5 min, incubated with peroxidase-conjugated rabbit anti-horse IgG (Thermo Fisher Scientific, Waltham, MA, USA) for 30 min, and again washed with TBS. Detection was performed using liquid DAB. Sections were counterstained with haematoxylin. Immunohistochemical expression was observed by optical microscopy (40 ×), with 100 nuclei counted in 10 fields; every distinct brown stain was considered positive [37].

### Histopathological analyses

A trained pathologist microscopically analysed H&E-stained slides. Specimens were examined and staged according to the guidelines of the Joint American Cancer Commission/International Union against Cancer/Classification of Malignant Tumours [38]. Metastatic disease was confirmed when, according to clinical records, lesions were detected in other organs or lymph nodes. The histological type of the tumour was classified as intestinal or diffuse [3].

### Statistical analysis

Data analysis was performed using SPSS 20.0 (IBM, Corp., Armonk, NY, US). Categorical variables are presented as frequencies and percentages. We used the logistic regression model and odds ratio (OR) and calculated the respective 95% confidence intervals (CI). Variable selection was conducted in a stepwise manner and the unadjusted and adjusted OR were considered in the final model. The goodness of fit was verified by a Hosmer–Lemeshow test. Only significant variables (*P* < 0.05) were used in the final model unless the variable was biologically relevant. A standard Chi-square test was used to assess the strength of association between the presence of a study microorganism and certain clinicopathological characteristics. The non-parametric Mann–Whitney U test was used to evaluate the microbial load of *M. hyorhinis* in the case and control groups (positive and negative), using GraphPad Prism version 5.01 (GraphPad Software, Inc., La Jolla, CA, USA).

## Results

### DNA extraction time, yield, and purity

Dewaxing of FFPEs using xylol required 30 to 90 min compared to 1 min with mineral oil. The phenol–chloroform (EM1, EM2, and EM3) extractions were more laborious and time-consuming than those performed using alkaline buffer (EM4) and commercial kits (EM5 and EM6) (Table 2). Table 2 also presents the mean values of the total amount and purity of extracted DNA. EM1, EM2, and EM6 showed the best results according to DNA quantity (2.12, 2.10, and 2.09, respectively) and purity ratios (1.91, 1.92, and 1.98, respectively). Based on our evaluation, all 120 FFPE samples were dewaxed with mineral oil and DNA was extracted using EM6.

**Table 2:** Evaluation of purity and amount of DNA extracted from fragments of FFPE gastric tissue samples

DNA extraction method	260/230*	260/280*	DNA (ng/µL)
*EM1*			
Xylol dewaxing material	2.12	1.91	2.507
Non-dewaxing material	2.35	1.75	47
*EM2*			
Xylol dewaxing material	2.10	1.92	475
Non-dewaxing material	2.47	1.66	168
*EM3*			
Xylol dewaxing material	1.82	1.59	99
Non-dewaxing material	1.59	1.56	53
*EM4*			
Xylol dewaxing material	0.38	1.19	800
Non-dewaxing material	0.38	1.27	431
*EM5*			
Xylol dewaxing material	1.38	1.81	243
Mineral oil dewaxing material	1.24	1.94	112
Non-dewaxing material	1.69	2.06	183
*EM6*			
Xylol dewaxing material	2.50	2.60	21
Mineral oil dewaxing material	2.09	1.98	82
Non-dewaxing material	2.96	2.78	16

*The wavelengths of 260/230 nm (ratio: 2.0–2.2) and 260/280 nm (ratio: 1.7–2.0) were considered acceptable for evaluation

In the 260/230 nm wavelength, EM1 and EM2 extraction methods and EM6 dewaxed with mineral oil show good DNA extraction. EM3, EM4, and EM5 extractions show low absorbance values, indicating contamination of salts (e.g. EDTA). At 260/280 nm, EM1, EM2 (dewaxed), EM5, and EM6 (dewaxed with mineral oil) present sufficient DNA purity. EM2 (Non-dewaxing material), EM3, EM4, and EM6 (xylol dewaxing material and non-dewaxing material) present insufficient DNA purity, indicating protein contamination and other organic compounds

DNA extraction was validated by qualitative confirmation using PCR for the endogenous housekeeping control *β-globin*, with primers PC03/PC04 (110 bp). This reaction generates a small amplification product and is more suitable for DNA extracted from paraffin [22]. In total, 113 samples were positive for *β-globin* (case group, 73; control group, 40).

### DNA detection of Mollicutes, *M. hyorhinis, H. pylori*, and *F. nucleatum* according to patient gender and age

Considering both the case and control groups, the mean patient age was 60 years (n = 120, Table 3), with a majority of male subjects (~ 60%). The case group consisted of predominantly males (~ 74%) and the control group of females (70%). Males had a ~ fivefold higher risk (CI = 1.647–17.239) of developing gastric cancer (*P* = 0.004). Based on the median age, patients above 59 years of age had a 32-fold greater risk (CI = 8.112–129.376) of developing gastric cancer (*P* = 0.001). Table 3 also presents the presence and absence of our bacterial study species, PCR was performed to detect the DNA of Mollicutes in all the samples positive for the *β-globin* gene (n = 113); 10 samples were found to be positive for Mollicutes (8.8%), nine from patients with cancer (12.3%) and one from a healthy patient (2.5%). The presence of Mollicutes did not affect the development of gastric cancer, but was associated with a ~ twofold higher cancer risk factor compared with the control group.

**Table 3:** Relationship between gender, age, and detection of microorganisms

Variables	Case n = 80 (%)	Control n = 40 (%)	Total n = 120 (%)	OR (Crude)		OR (Adjusted)^a^	
				*P* (Wald’s Test)	OR (95% CI)	*P* (Wald’s Test)	OR (95% CI)
*Gender*							
Male	59 (73.8)	12 (30.0)	71 (59.2)	0.001	6.556	0.005	5.328
Female	21 (26.2)	28 (70.0)	49 (40.8)		(2.831–15.183)		(1.647–17.239)
*Age*							
≥ 59 years	62 (77.5)	4 (10.0)	66 (55.0)	0.001	31.000	0.000	32.395
≤ 58 years	18 (22.5)	36 (90.0)	54 (45.0)		(9.731–98.753)		(8.112–129.376)
*Mollicutes (PCR)*							
Positive	9 (12.3) ^b^	1 (2.5)	10 (8.8) ^c^	0.563 ^c^	2.345	0.622 ^c^	2.294 (0.084–62.384) ^c^
Negative	64 (87.7) ^b^	39 (97.5)	103 (91.2) ^c^		(0.131–41.962) ^c^		
*H. pylori* (PCR)							
Positive	6 (8.2) ^b^	9 (22.5)	15 (13.3) ^c^	0.763 ^c^	0.671	0.978 ^c^	1.046
Negative	67 (91.3) ^b^	31 (77.5)	98 (86.7) ^c^		(0.050–8.946) ^c^		(0.042–25.885) ^c^
*F. nucleatum* (qPCR)							
Positive	19 (26.0) ^b^	1 (2.5)	20 (16.7) ^c^	0.080 ^c^	0.353	0.023^c^	0.130
Negative	54 (74.0) ^b^	39 (97.5)	100 (83.3) ^c^		(0.353–0.110) ^c^		(0.022–0.756) ^c^
*M. hyorhinis* (qPCR)							
Positive	8 (11.0) ^b^	2 (5.0)	10 (8.8) ^c^	0.021 ^c^	11.357	0.045 ^c^	10.562
Negative	65 (89.0) ^b^	38 (95.0)	103 (91.2) ^c^		(1.1443–89.397) ^c^		

Positive—Detection of expected DNA product. Negative—Absence of expected DNA product.

^a^ adjusted for *M. hyorhinis* and Mollicutes. The goodness of the adjustment was evaluated by the Hosmer–Lemeshow test (*P* = 0.914)

^b^ Considering the case group, n = 73

^c^ Considering n = 113 patients

OR, odds ratio; CI, confidence interval; qPCR, quantitative polymerase chain reaction

For *H. pylori*, 15 samples were positive (13.3%): six from patients with cancer (8.2%) and nine from healthy patients (22.5%). The presence of *H. pylori* did not affect the cancer risk factor in the case group. *H. pylori* was strongly associated with the control group (*P* = 0.023), which suggests that the stomach becomes a favourable environment for *H. pylori* infection after bariatric surgery (OR = 0.130, CI 95% = 0.022–0.756). For *F. nucleatum*, 20 samples were found to be positive (16.7%): 19 from patients with cancer (26.0%) and one from a healthy patient (2.5%) (Table 3). The prevalence of *F. nucleatum* in the case group was significant (*P* = 0.045)) and was associated with an 11-fold increase in the risk of developing gastric cancer (CI 95% = 1.057–105.521).

None of the samples tested positive for *M. hyorhinis* when using EM6 (data not shown). However, based on the qPCR results, eight samples from the case group (11.0%) and two samples from the control group (5.0%) were positive for *M. hyorhinis* (Table 3).

**Fig. 1:**
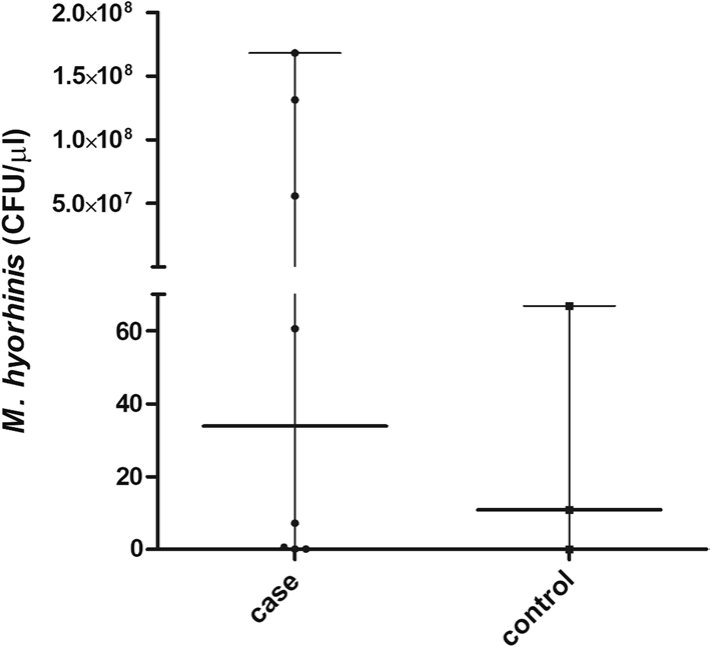
Quantification of *M. hyorhinis* by qPCR in formalin-fixed paraffin-embedded (FFPE) gastric tissue samples. The data did not follow a normal distribution, thus the median and range have been presented for the case group (33.9 CFU/mL, minimum and maximum load of 0.14–1.68 × 10^8^) and control group (10.9 CFU/mL, minimum and maximum load of 0.0–66.84). Statistical analysis performed by Mann–Whitney U, assuming significance at *p* < 0.05. qPCR, quantitative polymerase chain reaction

By employing a quantitative real-time PCR method, it was possible to quantify DNA as well as the microorganism load, represented as colony-forming units (CFU)/mL. The average loads of positive samples were approximately 10^0^ to 10^8^ CFU/mL of *M. hyorhinis* (Fig. 1). In the case group, the mean load of *M. hyorhinis* was 4.44 × 10^7^ CFU/mL (SD ± 6.85 × 10^7^_,_ minimum and maximum load of 0.14–1.68 × 10^8^) and in the control group the mean load was 38.86 CFU/mL (SD ± 35.87, minimum and maximum load of 0.0–66.84). We could not detect a significant difference in microbial loads between the groups. There was no association between the OR (epidemiological association between outcome variables [patients with cancer × patients without cancer]) and *M. hyorhinis* detection rates (presence × absence) (OR = 0.671; CI = 0.050–8.946; Table 3).

### Immunohistochemical detection of *M. hyorhinis* in FFPE samples of gastric tissues

**Fig. 2:**
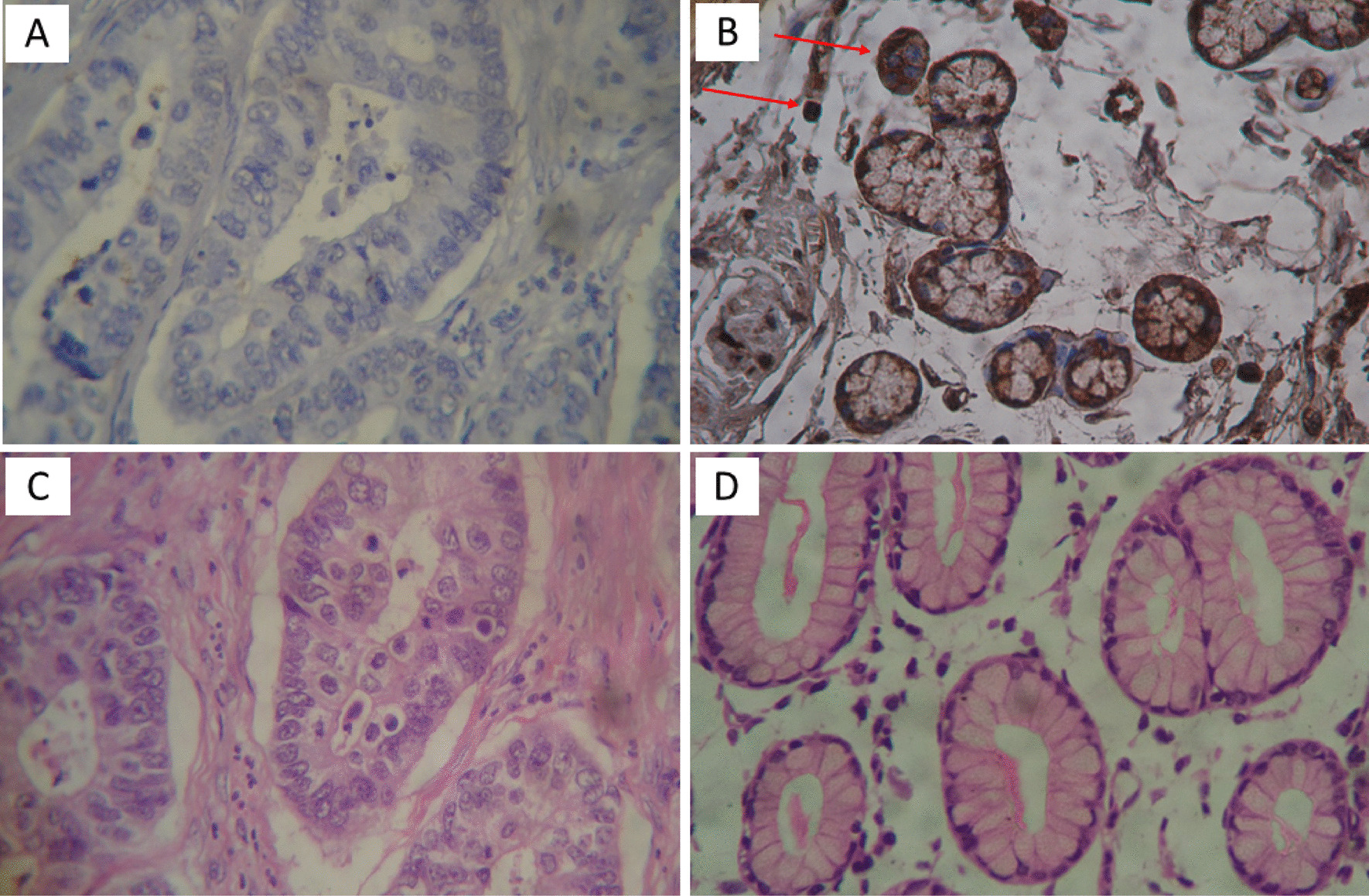
- Micromorphology of immunohistochemical (IHC) examination of gastric tissue treated with antibodies against *M. hyorhinis* protein p37. **A** Unmarked (negative). **B** Strong marking (positive). Arrows indicate the positive immunoreactivity for *M. hyorhinis*, with well-coloured cytoplasm. **C** and **D** haematoxylin–eosin (H&E)-stained sections. Gastric adenocarcinoma at 40 × magnification

IHC was used to confirm the presence of *M. hyorhinis* in 120 samples prepared on slides using anti-*M. hyorhinis* antibodies (Fig. 2). Eight samples were positive for *M. hyorhinis* (6.7%), all belonging to the case group (n = 80).

**Fig. 3:**
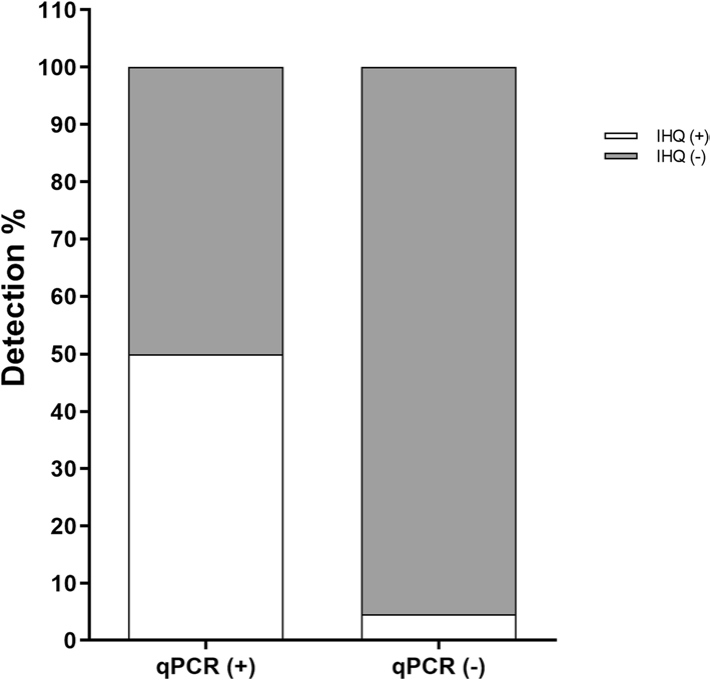
Detection of *M. hyorhinis* by IHC and qPCR in gastric cancer tissues. In the case group (n = 73), eight patients were found to be positive for *M. hyorhinis* by qPCR and, among these, four were found to be positive by IHC detection (50%). In total, 65 samples were negative for *M. hyorhinis* by qPCR; among these, three were positive for *M. hyorhinis* by IHC detection (4.6%). qPCR, quantitative polymerase chain reaction

The reactivity of the IHC technique was compared with the qPCR technique. We evaluated 73 samples by qPCR of which 50% tested positive following IHC detection (Fig. 3). Interestingly, a patient who was not examined using molecular techniques was found to be positive by IHC.

### Detection of mollicutes, *M. hyorhinis, H. pylori*, and* F. nucleatum* and associations with clinical factors in the case group

Clinical and pathological factors and their association with microorganisms were compared (Table 4). The presence of Mollicutes showed a strong relationship with papillary arrays (*P* = 0.003). No statistically significant association was observed between the presence of *M. hyorhinis* and clinicopathological data. The detection of *H. pylori* was associated with metastasis (*P* = 0.024), lymphatic invasion (*P* = 0.033), diffused tumour (*P* = 0.028), and the GX/G1/G2 histopathological grade (*P* = 0.008). The histopathological grading of cancer is classified as GX (where it was not possible to evaluate the degree of differentiation), G1 (well-differentiated), and G2 (moderately differentiated). The detection of *F. nucleatum* was associated with advanced age > 59 (*P* = 0.030) and large tumour fragments > 4 cm (*P* = 0.053).

**Table 4:** Associations between the presence of Mollicutes, *H. pylori*, *F. nucleatum* and *M. hyorhinis* and clinicopathological factors

Clinicopathological characteristics	Cases n = 73 (%)	*Mollicutes (%)*		*H. pylori (%)*		*F. nucleatum (%)*		*M. hyorhinis (%)*			
		PCR Positive	*P*	PCR Positive	*P*	qPCR Positive	*P*	qPCR Positive	*P*	IHC Positive	*P*
*Gender*											
Male	54 (74.0)	6 (11.1)	0.781	4 (7.4)	0.670	12 (22.2)	0.142	4 (7.4)	0.102	5 (8.5) ^a^	0.446 ^*a*^
Female	19 (26.0)	3 (15.8)		2 (10.5)		4 (21.1)		4 (21.1)		3 (14.3) ^a^	
*Age*											
≤ 59	18 (24.7)	1 (5.6)	0.553	2 (11.1)	0.607	4 (22.2)	0.030	1 (5.6)	0.398	0 (0.0) ^a^	0.108 ^a^
> 59	55 (75.3)	8 (14.5)		4 (7.3)		15 (27.3)		7 (12.7)		8 (12.9) a	
*Tumour size (cm)*											
≤ 4	24 (32.9)	4 (16.7)	0.430	2 (8.3)	0.980	6 (25.0)	0.053	3 (12.5)	0.768	4 (14.8) ^a^	0.306 ^a^
> 4	49 (67.1)	5 (10.2)		4 (8.2)		13 (26.5)		5 (10.2)		4 (7.5) ^a^	
*TNM staging*											
I/II	20 (27.4)	3 (15.0)	0.670	1 (5.0)	0.538	2 (10.0)	4.040	3 (15.0)	0.497	2 (8.7) ^a^	0.805 ^a^
III/IV	53 (72.6)	6 (11.3)		5 (9.4)		17 (32.1)		5 (9.4)		6 (10.5) ^a^	
*Metastasis*											
Positive	41 (56.2)	7 (17.1)	0.163	6 (14.6)	0.024	12 (29.3)	0.326	6 (14.6)	0.255	5 (10.9) ^a^	0.763 ^a^
Negative	32 (43.8)	2 (6.3)		0 (0.0)		7 (21.9)		2 (6.3)		3 (8.8) ^a^	
*Regional lymph nodes*											
NX	3 (4.1)	0 (0.0)	0.476	0 (0.0)	0.196	0 (0.0)	4.336	0 (0.0)	0.366	2 (50.0) ^a^	0.064 ^a^
N0	28 (38.4)	2 (7.1)		0 (0.0)		6 (21.4)		2 (7.1)		2 (6.9) ^a^	
N1	26 (35.6)	3 (11.5)		4 (15.4)		8 (30.8)		2 (7.7)		2 (6.7) ^a^	
N2	12 (16.4)	3 (25.0)		2 (16.7)		5 (41.7)		3 (25.0)		1 (7.7) ^a^	
N3	4 (5.5)	1 (25.0)		0 (0.0)		0 (0.0)		1 (25.0)		1 (25.0) ^a^	
*Lymphatic invasion*											
Positive	43 (58.9)	7 (16.3)	0.219	6 (13.9)	0.033	13 (30.2)	0.736	6 (13.9)	0.327	4 (8.3) ^a^	0.543 ^a^
Negative	30 (41.1)	2 (6.7)		0 (0.0)		6 (20.0)		2 (6.7)		4 (12.5) ^a^	
*Vascular invasion*											
Positive	11 (15.1)	2 (18.2)	0.522	2 (18.2)	0.192	6 (54.5)	3.421	1 (9.1)	0.830	2 (14.3) ^a^	0.556 ^a^
Negative	62 (84.9)	7 (11.3)		4 (6.5)		13 (21.0)		7 (11.3)		6 (9.1) ^a^	
*Tumour type (Lauren's Class.) *^*3*^											
Intestinal	39 (53.4))	2 (5.1)	0.110	2 (5.1)	0.028	6 (15.4)	2.609	3 (7.7)	0.564	1 (2.6) ^a^	0.077 ^a^
Diffuse	33 (45.2	7 (21.2)		3 (9.1)		13 (39.4)		5 (15.2)		6 (18.2) ^a^	
*Histopathological grade*											
GX	1 (1.4)	0 (0.0)	0.109	1 (100.0)	0.008	10 (25.6)	1.331	0 (0.0)	0.649	0 (0.0) ^a^	0.892 a
G1	39 (53.4)	2 (5.1)		2 (5.1)		0 (0.0)		3 (7.7)		4 (8.9) ^a^	
G2	31 (42.5)	6 (19.4)		3 (9.7)		9 (29.0)		5 (16.1)		4 (12.5) ^a^	
G3	2 (2.7)	1 (50.0)		0 (0.0)		0 (0.0)		0 (0.0)		2 (100.0) ^a^	
G4	0 (0.0)	0 (0.0)		0 (0.0)		0 (0.0)		0 (0.0)		0 (0.0) ^a^	
*Haemorrhage*											
Positive	30 (41.1)	3 (10.0)	0.395	3 (10.0)	0.861	8 (26.7)	0.084	3 (10.0)	0.586	4 (13.3) ^a^	0.764 ^a^
Negative	43 (58.9)	6 (14.0)		3 (16.3)		11 (25.6)		5 (11.6)		4 (9.3) ^a^	
*Inflammation*											
Positive	17 (23.3)	3 (17.6)	0.446	3 (17.6)	0.106	5 (29.4)	0.072	2 (11.8)	0.903	2 (8.7) ^a^	0.805 ^a^
Negative	56 (76.7)	6 (10.7)		3 (5.4)		14 (25.0)		6 (10.7)		6 (10.5) ^a^	
*Presence of eosinophils*											
Positive	13 (17.8)	3 (23.1)	0.194	2 (15.4)	0.299	4 (30.8)	0.087	2 (15.4)	0.573	1 (6.7) ^a^	0.633 ^a^
Negative	60 (82.2)	6 (10.0)		4 (6.7)		15 (25.0)		6 (10.0)		7 (10.8) ^a^	
*Presence of neutrophils*											
Positive	14 (19.2)	2 (14.3)	0.804	2 (14.3)	0.358	2 (14.3)	2.049	1 (7.1)	0.611	1 (5.6) ^a^	0.475 ^a^
Negative	59 (80.2)	7 (11.9)		4 (6.8)		17 (28.9)		7 (11.9)		7 (11.3) ^a^	
*Presence of macrophages*											
Positive	17 (23.3)	0 (0.0)	0.078	0 (0.0)	0.159	3 (17.6)	0.872	1 (5.9)	0.444	0 (0.0) ^a^	0.096 ^a^
Negative	56 (76.7)	9 (16.1)		6 (10.7)		16 (28.6)		7 (12.5)		8 (13.1) ^a^	
*Tissue necrosis*											
Positive	16 (21.9)	4 (25.0)	0.081	0 (0.0)	0.176	5 (31.3)	0.621	2 (12.5)	0.823	3 (18.8) ^a^	0.192 ^a^
Negative	57 (78.1)	5 (8.8)		6 (10.5)		14 (24.6)		6 (10.5)		5 (7.8) ^a^	
*Papillary arrangements*											
Positive	6 (8.2)	3 (50.0)	0.003	0 (0.0)	0.444	0 (0.0)	2.389	1 (16.7)	0.640	2 (28.6) ^a^	0.086 a
Negative	67 (91.8)	6 (9.0)		6 (9.0)		19 (28.4)		7 (10.4)		6 (8.2) ^a^	
*Tumour location*											
Cardia	4 (5.5)	1 (25.0)	0.703	0 (0.0)	0.365	0 (0.0)	1.698	1 (25.0)	0.759	0 (0.0) ^a^	0.881 ^a^
Body	25 (34.2)	4 (16.0)		4 (16.0)		6 (24.0)		2 (8.0)		3 (12.0) ^a^	
Antrum	43 (58.9)	4 (9.3)		2 (4.7)		13 (30.2)		5 (11.6)		5 (10.0) ^a^	
Body and Antrum	1 (1.4)	0 (0.0)		0 (0.0)		0 (0.0)		0 (0.0)		0 (0.0)	

^a^ Considering case group, n = 80

qPCR, quantitative polymerase chain reaction; IHC, immunohistochemistry

### Coinfection profile and detection rate of mollicutes, *M. hyorhinis*, *F. nucleatum*, and *H. pylori* in the case and control groups

**Fig. 4:**
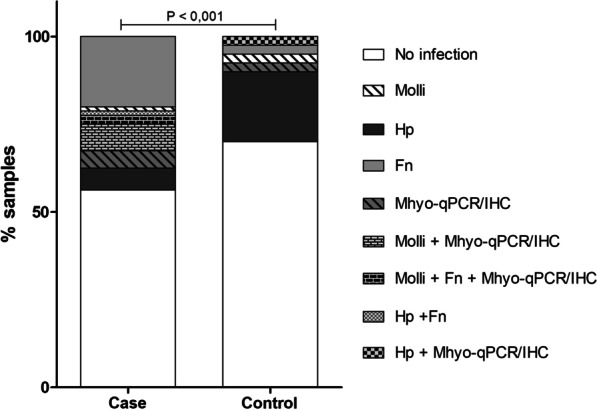
Detection rate of Mollicutes (PCR), *He. pylori* (PCR), *F. nucleatum* (qPCR), and *M. hyorhinis* (qPCR and IHC), from gastric tissue. The diagonal lines on the white background represent Mollicutes (Molli) in the case and control group (1.25% and 2.50%, respectively). Dark-grey represents *H. pylori* (Hp) in the case and control group (6.25% and 20.00%, respectively). Light-grey represents *F. nucleatum* (Fn) in the case and control group (20.00% and 2.50%, respectively). The diagonal lines on the grey background represent *M. hyorhinis* (Mhyo-qPCR/IHC) in the case and control group (5.00% and 2.50%, respectively). Coinfection of the case samples: 7.50% with Mollicutes and *M. hyorhinis* (brick with white background), 2.50% of Mollicutes (*M. hyorhinis*) and *F. nucleatum* (brick with black background), and 1.25% of *F. nucleatum* and *H. pylori* (light-grey squares). In the control samples, only one coinfection of *M. hyorhinis* and *H. pylori* was observed (2.50%, dark-grey squares). qPCR, quantitative polymerase chain reaction; IHC, immunohistochemistry. Mann Whitney, assuming *p*-value ≤ 0.05

There was no difference in the abundance of Mollicutes, *M. hyorhinis*, or *F. nucleatum* between the case and controls groups (*P* = 0.117, *P* = 0.140, and *P* = 0.214, respectively). However, the abundance of *H. pylori* differed significantly between the case and control groups (*P* = 0.008). Coinfection profiles, the detection of two or more study microorganisms in the same clinical sample, were analysed in the two groups (Fig. 4). In the case group samples that were positive for Mollicutes (n = 73), six were positive for *M. hyorhinis* according to qPCR and IHC, respectively. The only sample positive for Mollicutes in the control group was negative for *M. hyorhinis* according to PCR. Mollicutes, *M. hyorhinis*, and *F. nucleatum* showed coinfection in two samples of the case group alone.

*H. pylori* and *F. nucleatum* showed coinfection only in one sample of the case group. *H. pylori* showed no coinfection with Mollicutes, but in the control group (n = 40) there was coinfection with *M. hyorhinis* and *H. pylori* in one patient. Overall, 56.25% of the case group samples and 70.00% of the control group samples were negative for any of the study microorganisms. The detection of *H. pylori* was higher in the control group, likely due to the favourable conditions created by congestion in the gastric wall that promotes acid secretion and inflammation. There was a significant difference in microorganism abundance between the groups (*P* = 0.001). Unlike *H. pylori*, the detection frequency of Mollicutes, *F. nucleatum*, and *M. hyorhinis* were considerably higher in the case group.

## Discussion

Cancer has been a major research focus worldwide, with diverse risk factors associated with its development. In Brazil, stomach cancer mortality rates are male-biased, with the highest rates of occurrence observed in the Northeast region [2]. Brazil and other developing countries are disproportionately affected by infectious agents that are considered risk factors for developing cervical, liver, and stomach cancer [1]. Research into *M. hyorhinis* gained widespread momentum after its detection in human carcinomas and can potentially induce cell migration and metastasis, correlating with changes in phenotype and cellular karyotype. This change in cellular behaviour was associated with protein p37, present in its cell membrane [18, 39]. In general, mycoplasmas have been associated with malignant transformation in cell cultures and immortalisation of cells, promoting cell migration/invasion in immunosuppressed mice [4, 40]. Likewise, *F. nucleatum* has also been reported to cause tumour progression in immunosuppressed mice subjected to xenografts of patient-derived tumours [41].

The storage of tissues in paraffin blocks is convenient for transport, and also allows for retrospective examinations that could provide valuable information in epidemiology [22]. However, the recovery of nucleic acids in this type of material is difficult and with no methodological consensus in the literature with regards to optimal procedures. In the present study, six methods for DNA extraction from gastric fragments in FFPE blocks were compared for the detection of Mollicutes, *M. hyorhinis, F. nucleatum*, and *H. pylori*. Mineral oil dewaxing and commercial kits demonstrated the desired preferences, based on elapsed time, absence of toxic organic solvent reagents (such as phenol), and quality of target DNA after PCR [25, 27, 42]. Although extraction kits obtained a smaller amount of DNA, they allowed for a more efficient detection of targeted DNA, as residues that inhibit PCR efficacy are eliminated, thus reducing the possibility of sample contamination [43].

The DNA extraction methodology that demonstrated greater purity and recovery of nucleic acids was used for detecting the *β-globin* gene with PC03/PC04 primers, which was identified in 113 gastric tissue samples. FFPE samples are usually exposed to formaldehyde for a prolonged period of time, which modifies protein morphology and DNA stability and, additionally, causes DNA fragmentation; hence, successful detection of housekeeping genes increases with smaller products owing to the increased possibility of amplifying fragmented DNA [22, 29, 33].

Mollicutes were detected in only 8.8% of the samples (10/120): 12.3% of the case group (9/73) and 2.5% of the control group (1/40), indicating a low prevalence of these microorganisms in FFPEs. This low detection rate does not rule out the possibility that the Mollicutes contribute to cellular changes in cancer, since detection can be affected by the host's immune response. The use of antibiotics to treat an early-stage *H. pylori* infection can also suppress Mollicutes, and *H. pylori* itself is a strong competitor that can inhibit the proliferation of other microorganisms [44, 45]. In the present study, Mollicutes were not detected in individuals with *H. pylori* and gastric cancer, though this could be an artefact of small sample size.

Intestinal and diffuse gastric tumours with similar clinical characteristics have been associated with greater metastatic potential, rapid tumour progression, and poor prognoses, which is induced by chromosomal aberrations and papillary tumours [40, 41]. The presence of Mollicutes was predominant in the diffuse type tumour, in moderately differentiated tumours (G2), and in patients with metastases. Although this association was not significant, *Mycoplasma* are considered opportunistic pathogens and cytogenetic modifiers [42, 43], with the ability to persist in the host for a prolonged period of time, favouring the gradual dysregulation of cell biology [46].

The Mollicutes showed a strong association with papillary tissue arrays (8.2% of patients). Previous research has also found Mollicutes associated with polyp formations in colon and mycoplasma infections [16]. Research suggests that *Mycoplasma* is able to stimulate cell transformation [11, 46]; production of lipid-associated membrane proteins (LAMPs) or macrophage-activating lipopeptide-2 (MALP-2), which induce the MyD88 pathway and NF-κB activation through toll-like receptors 1, 2, and 6 [47, 48]; induce major inflammatory events via nitric oxide synthase (iNOS) [48, 49]. Cell aberrations induced during *Mycoplasma* infection may also originate from their capacity to block host DNA repair, as seen in in vitro assays with the protein DnaK, isolated from *M. fermentans*. This chaperone binds to poly (ADP-ribose) polymerase (PARP-1) or in the p53 regulator, impairing the host's DNA repair capacity and leading to p53 silencing [50]. Moreover, p53 silencing may stimulate chromosomal instability and cell transformation [40, 51]. Blocking of the host's DNA repair capacity occurs during *H. pylori* infection, which induces NF-κB expression, leading to chronic inflammation and atrophic gastritis, metaplasia, and dysplasia [20, 52].

Persistent *H. pylori* infection has been classified as carcinogenic to humans (Group 1) by the International Agency for Research on Cancer [53]. In the present study, *H. pylori* infection was detected in control individuals, which can be expected in a stomach microenvironment altered by surgery and can trigger the development of ulcers, making this site even more favourable for the establishment of this bacteria [54, 55]. The clinical aspects of infection vary depending on location, host susceptibility, bacterial strain (not all strains of *H. pylori* are oncogenic), environmental influence, and host habits [56]. Approximately 10% to 15% of individuals with *H. pylori* infection may develop severe inflammation and peptic ulcers [57], as well as gastric adenocarcinoma (1–3%) or lymphoma (0.1%) [58].

In our study, *H. pylori* was significantly associated with metastasis (*P* = 0.024) and lymphatic invasion (*P* = 0.033). In a pervious study, *H. pylori* has been associated with the expression of CagA, the most important virulence factor of *H. pylori*, which induces the robust activation of SHP-2 and ERK. CagA also promotes tumorigenesis by inducing gastric epithelial cell mobility and proliferation [59]. In the present study, *H. pylori* was located in non-cardia regions of the stomach, similar to previous studies [60]. The presence of *H. pylori* was marginally associated with differentiated (G2) (*P* = 0.008) and diffused type (*P* = 0.028) tumours. These tumour phenotypes have also been correlated with *H. pylori* in a previous study [61]. Moreover, *H. pylori* has also been associated with the dysregulation of pepsinogen I (PG1) and pepsinogen 2 (PG2) [62], decreased gastric glands [63], and expression of interleukin (IL)-10, leading to an anti-inflammatory response that attenuates the immune system's response [64].

The silencing of the human immune response against tumorigenesis has also been reported during *F. nucleatum* infection, that contributes to carcinogenesis by inducing preneoplastic conditions [9]. Although *F. nucleatum* is predominant in colorectal tumours, it is also well established in gastric tumour tissues, where it is associated with poor survival rates by promoting metastasis and diffuse-type tumour progression [65]. In the present study, *F. nucleatum* was predominant in the stomach and body and antrum regions in patients with metastasis and lymphatic invasion, similar to *H. pylori*. However, *H. pylori* is reportedly an initiator of the Correa cascade and *F. nucleatum* is not related to chronic gastritis, so rather than being an initiator, *F. nucleatum* may be involved in the final stages of the classical Correa cascade [65].

Diffuse-type tumours are strongly associated with E-cadherin dysregulation. It is well known that *F. nucleatum* promotes carcinogenesis by linking FadA to E-cadherin, activating β-catenin and the Wnt pathway, inducing the expression of several oncogenic properties [66]. FadA expression, in addition to being associated with E-cadherin expression, is also strongly associated with host DNA damage, cell proliferation, chk2 expression, S-phase cells, and an increase in tumour size [66]. In our study, the presence of *F. nucleatum* was significant at tumour size > 4 cm (*P* = 0.053). Tumour size and TNM stage III/IV have also been associated with *F. nucleatum* in other studies [67]. In addition to FadA mechanisms, TLR4 activation by *F. nucleatum*’ LPS can plausible lead to the activation of Myd88 and NF-κB, which leads to oncogenic overexpression of miR21, resulting in dysregulated growth and infiltration of tumour tissue. According to our results, the presence of *F. nucleatum* was significant in patients > 59 years of age (*P* = 0.30), which could be due to lower global long interspersed element-1 (LINE-1) DNA methylation [65], and epigenetic silencing of the MLH1 repair protein caused by cytokine modulation (e.g., IL-6 e TNF) [68]. This suggest that *F. nucleatum* may be associated with epigenetic alterations, such as global DNA hypomethylation. Similarly, *M. hyorhinis* has been described as an intracellular agent that could modulate the host epigenetic machinery through nucleomodulins [69].

A possible mechanism of tumour pathogenicity of *M. hyorhinis* may be due to the activity of its methyltransferases, in which three types have already been described: Mhy1 and Mhy2, which promote methylation of CG, and Mhy3, which acts on GATC sites leading to aberrant methylation patterns in its entire genome [69]. Hypermethylation of the GATC site is related to the silencing of specific genes, such as *APC*, *TP53*, *BRCA2*, *KRAS*, *PTEN*, *SMAD4*, and *VEGFC*. GATC methylation is uncommon in human cells and may emerge during *M. hyorhinis* infection, suggesting a new type of epigenetic infection-dependent marker [69]. Other mycoplasmas, such as *M. penetrans* and *M. pulmonis*, can also methylate GC sites, indicating that methylation may be a significant factor for these bacteria to adapt and survive in the host [70]. Aberrant methylation frequently contributes to malignant cellular phenotypes [4]. Furthermore, *H. pylori* infection can induce the aberrant methylation of genes such as tp53 and the Wnt pathway—factors strongly associated with gastric adenocarcinoma development [71]. Similar to *H. pylori*, *M. hyorhinis* was more biased toward the G2 tumour and diffused type, unlike what has been found in previous studies [17], and was localised mainly in the stomach body. This adenocarcinoma mainly affects older adults and can lead to a permanent inflammatory process and the gradual alteration of cells [64].

*M. hyorhinis* could not be detected by conventional PCR, probably because its PCR product was too large and DNA from paraffin specimens tends to come in small fragments [27]. According to qPCR analysis, 11.0% of the case group samples were positive for *M. hyorhinis*; among these, six were paired with the positive Mollicutes PCRs. The association of *M. hyorhinis* with gastric cancer development has previously been reported in studies showing that 50% of FFPE gastric cancer samples were positive for *M. hyorhinis* by PCR or IHC detection targeting p37 [16, 72]. qPCR also detected the DNA of *M. hyorhinis* in 5.0% of the samples from the control group. However, the samples that were positive for *M. hyorhinis* did not match the positive samples for Mollicutes. Indeed, other species of the class Mollicutes have been described in samples of gastric disease [11].

The detection of *M. hyorhinis* by the IHC revealed that ~ 10% of the samples in the case group were positive. In the case group, 8 samples positive for *M. hyorhinis* were detected, 4 of which were detected by both the IHC and qPCR methods. In the control group, two samples were positive for *M. hyorhinis* and were detected by qPCR, whereas IHC failed to detect the protein p37. The IHC could be a better method for target detection when considering that the formaldehyde in blocks may damage the *M. hyorhinis* DNA composed of approximately 74% A + T bases, while formalin preserves the protein morphology and inactivates proteolytic enzymes that favour the IHC methodology. Nevertheless, the qPCR method showed a higher detection sensitivity, indicating that the minimal amounts of DNA remain intact even under the action of chemical solutions and storage time; truncated proteins can also be difficult to detect by IHC [73]. These differences between tests have been observed in other studies [37]. The samples positive for *M. hyorhinis* by IHC were associated with gastric cancer risk, as reported in previous investigations [16].

If the detection of *M. hyorhinis* by IHC and qPCR was possible, then we assume that the assessed individuals were exposed to this microorganism at some level. Our current understanding of how *M. hyorhinis* infects humans remains poor, though pathogenic and environmental factors undoubtedly play a role. Indeed, the opportunistic character of Mollicutes, mainly antigenic variation, oxidative stress induction, and a limited host immune response, allow these bacteria to establish permanent infections and settle in different ecological niches [46]. Several Mollicute species are typically found in animals but have been detected in humans without symptoms of disease, though some species may cause disease, particularly in immunocompromised individuals [10].

In the present study, *M. hyorhinis* was detected in gastric samples belonging to 59 patients, presenting tumour invasion (TNM stage III/IV) and lymphatic invasion. The *M. hyorhinis* p37 membrane protein is described to interact with TLR4 and induce the rapid expression of several genes linked to inflammation, in addition to promoting the progression of cancer toward metastasis, as it facilitates PI3k phosphorylation and activation of the PI3k-AKT pathway [21, 46]. The p37 protein induces the expression of HRAS (known capacity in mycoplasmas), resulting in PI3k phosphorylation, which regulates cell motility [74]. In prostate cells, p37 has the ability to induce matrix metalloproteinase-2 (MMP-2) super activity and thereby increases EGFR phosphorylation, this can potentially result in cells acquiring a greater potential for dissemination, thus inducing metastasis [46]. This indicates that the ability of these microorganisms to colonise and promote inflammation in the gastric environment is strongly associated with the modulation of mechanisms expressed in local cells.

## Conclusion

This study was limited to exploring the clinicopathological characteristics of gastric tumour tissues and their associations with microorganisms, opening doors for the exploration of in vitro methods to analyse which microbial factors induce different cell signalling pathways, metabolic changes, or extracellular responses in cell matrix models.

Collectively, our findings indicate that FFPE tissue samples improved our previous understanding with regards to the strength of association of Mollicutes, *M. hyorhinis, F. nucleatum*, and *H. pylori* in gastric cancer. *H. pylori* alone may not lead to all the changes necessary for neoplastic development, but the complex feedback of the microbiotic community can contribute to the conditions of the disease. *F. nucleatum* is frequently found in biopsies of patients with gastric cancer, but there are still many unanswered questions about its role in the development of the disease.

Mollicutes are often underestimated as potential factors that drive diverse pathological aspects of certain diseases, but the association of mollicutes with the formation of papillary arrays demonstrates its importance in terms of observed malignant phenotypes. Mollicutes of animal origin represent a wide field of classical opportunistic bacteria capable of inducing human disturbances. The detection of *M. hyorhinis* in Brazilian populations with stomach cancer is an important finding. Therefore, this study contributes to a better understanding of the role of these bacteria in the development of gastric cancer and its malignant transformation.

## Data Availability

All data generated or analysed during this study are included in this article. The other raw datasets used and/or analysed in this study will be made available upon reasonable request to the corresponding author.

## Abbreviations

BA: Bahia
FFPE: Formalin-fixed paraffin-embedded
IHC: Immunohistochemical
H&E: Haematoxylin-eosin
EM: Extraction method
PK: Proteinase K
CFU: Colony-forming unit
GX: Not possible to evaluate the degree of tumour differentiation
G1: Tumour well-differentiated
G2: Tumour moderately differentiated
IARC: International Agency for Research on Cancer
